# CINeMA: An approach for assessing confidence in the results of a network meta-analysis

**DOI:** 10.1371/journal.pmed.1003082

**Published:** 2020-04-03

**Authors:** Adriani Nikolakopoulou, Julian P. T. Higgins, Theodoros Papakonstantinou, Anna Chaimani, Cinzia Del Giovane, Matthias Egger, Georgia Salanti

**Affiliations:** 1 Institute of Social and Preventive Medicine, University of Bern, Bern, Switzerland; 2 Population Health Sciences, Bristol Medical School, University of Bristol, Bristol, United Kingdom; 3 Université de Paris, Research Center of Epidemiology and Statistics Sorbonne Paris Cité (CRESS UMR1153), INSERM, INRA, Paris, France; 4 Cochrane France, Paris, France; 5 Institute of Primary Health Care (BIHAM), University of Bern, Bern, Switzerland

## Abstract

**Background:**

The evaluation of the credibility of results from a meta-analysis has become an important part of the evidence synthesis process. We present a methodological framework to evaluate confidence in the results from network meta-analyses, Confidence in Network Meta-Analysis (CINeMA), when multiple interventions are compared.

**Methodology:**

CINeMA considers 6 domains: (i) within-study bias, (ii) reporting bias, (iii) indirectness, (iv) imprecision, (v) heterogeneity, and (vi) incoherence. Key to judgments about within-study bias and indirectness is the percentage contribution matrix, which shows how much information each study contributes to the results from network meta-analysis. The contribution matrix can easily be computed using a freely available web application. In evaluating imprecision, heterogeneity, and incoherence, we consider the impact of these components of variability in forming clinical decisions.

**Conclusions:**

Via 3 examples, we show that CINeMA improves transparency and avoids the selective use of evidence when forming judgments, thus limiting subjectivity in the process. CINeMA is easy to apply even in large and complicated networks.

## Abstract 2

Adriani Nikolakopoulou and co-authors discuss CINeMA, an approach for evaluating the findings of network meta-analyses.

## Introduction

Network meta-analysis has become an increasingly popular tool for developing treatment guidelines and making recommendations on clinical effectiveness and cost-effectiveness. However, fewer than 1% of published network meta-analyses assess the credibility of their conclusions [1]. The Grading of Recommendations Assessment, Development and Evaluation (GRADE) approach provides an assessment of the confidence in the results from systematic reviews and meta-analyses, and many organisations, including the World Health Organization, have adopted the GRADE approach [2,3]. Based on GRADE, 2 systems have been proposed to evaluate the credibility of results from network meta-analyses [4,5], but the complexity of the methods and lack of suitable software have limited their uptake.

Here we introduce the Confidence in Network Meta-Analysis (CINeMA) approach. CINeMA is broadly based on the GRADE framework, with several conceptual and semantic differences [5]. It covers 6 domains: (i) within-study bias (referring to the impact of risk of bias in the included studies), (ii) reporting bias (referring to publication and other reporting bias), (iii) indirectness, (iv) imprecision, (v) heterogeneity, and (vi) incoherence. The reviewer’s input is required at the study level for within-study bias and indirectness. Then, applying user-defined rules, CINeMA assigns judgments at 3 levels (no concerns, some concerns, or major concerns) to each domain. Judgments across domains can be summarised to obtain 4 levels of confidence for each relative treatment effect, corresponding to the usual GRADE assessments of very low, low, moderate, or high.

We will focus on randomised controlled trials, and on relative treatment effects. We assume that evaluation of the credibility of results takes place once all primary analyses and sensitivity analyses have been undertaken. We further assume that reviewers have implemented pre-specified inclusion criteria for studies and have obtained the best possible estimates of relative treatment effects using appropriate statistical methods [6–9].

We illustrate the methods using 3 examples that highlight different aspects of the process and represent networks of different complexities. Two examples are introduced in Box 1: a network of trials that compare outcomes of diagnostic strategies in patients presenting with symptoms suggestive of acute coronary syndrome [10] and a network comparing adverse events of statins [11]. A third network, comparing the effectiveness of 18 antidepressants for major depression [12], is evaluated for all domains and is presented in S1 Text. All analyses were done in R software using the *netmeta* package and the CINeMA web application (Box 2) [13,14].

**Box 1. Description of 2 network meta-analyses used to illustrate the CINeMA approach to assess confidence in network meta-analysis.**

**Diagnostic strategies for patients presenting with symptoms suggestive of acute coronary syndrome**

**Fig 1:**
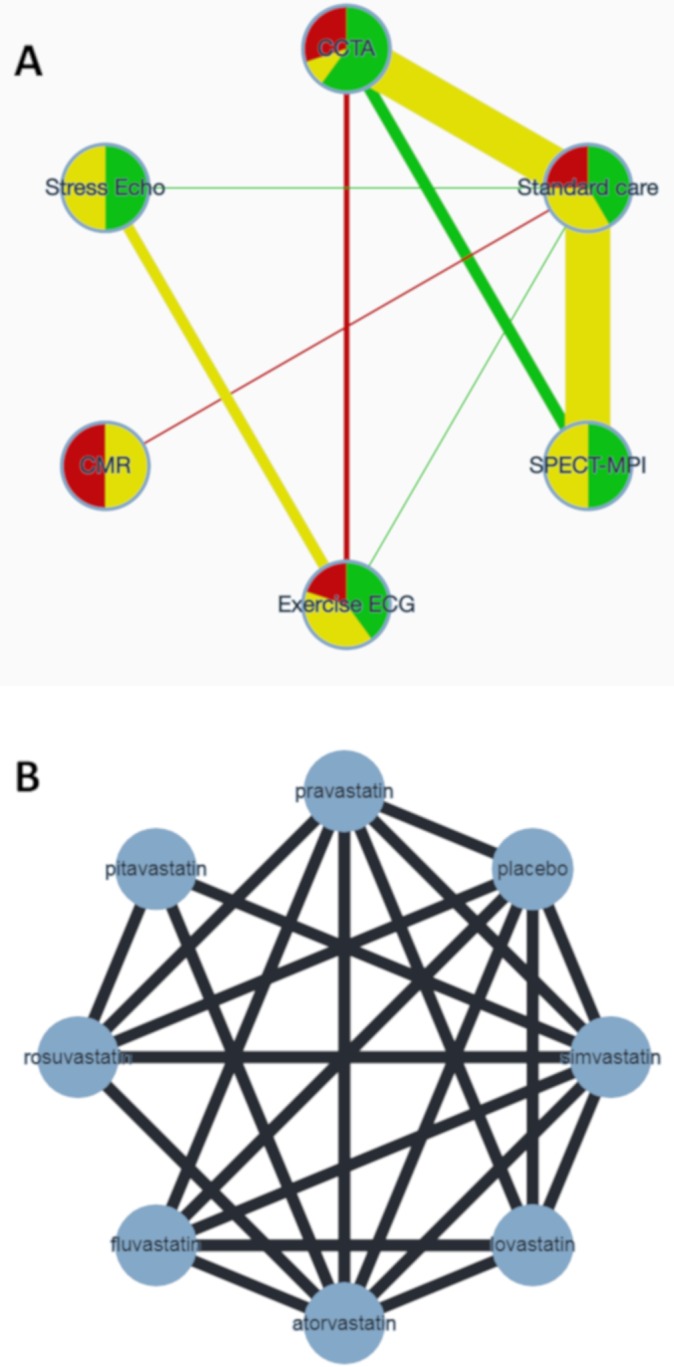
Network plots of 2 network meta-analyses. (A) Network of randomised controlled trials comparing non-invasive diagnostic strategies for the detection of coronary artery disease in patients presenting with symptoms suggestive of acute coronary syndrome. The width of the edges is proportional to the number of patients randomised in each comparison. The colours of edges and nodes refer to the risk of bias: low (green), moderate (yellow), and high (red). (B) Network of randomised controlled trials comparing statins with respect to adverse effects. CCTA, coronary computed tomography angiography; CMR, cardiovascular magnetic resonance; ECG, electrocardiogram; echo, echocardiography; SPECT-MPI, single photon emission computed tomography–myocardial perfusion imaging.

Siontis et al. performed a network meta-analysis of randomised trials to evaluate the differences between the non-invasive diagnostic modalities used to detect coronary artery disease in patients presenting with symptoms suggestive of acute coronary syndrome [10]. Differences between the diagnostic modalities were evaluated with respect to the number of downstream referrals for invasive coronary angiography and other clinical outcomes. For the outcome referrals, 18 studies were included. The network is presented in Fig 1A, and the data in S1 Data. The results from the network meta-analysis are presented in Table 1.

**Comparative tolerability and harms of statins**

The aim of the systematic review by Naci et al. [11] was to determine the comparative tolerability and harms of 8 statins. The outcome considered here was the number of patients who discontinued therapy due to adverse effects, measured as an odds ratio. This outcome was evaluated in 101 studies. The network is presented in Fig 1B, and the outcome data are given in S2 Data. The results of the network meta-analysis are presented in S1 Table, and the results from Separating Indirect from Direct Evidence (SIDE) in S2 Table.

**Table 1:** Results from pairwise (upper triangle) and network (lower triangle) meta-analysis from the network of non-invasive diagnostic strategies for the detection of coronary artery disease in Fig 1A.

CCTA	—	2.25	1.04	1.23	—
		(1.04–4.90)	(0.70–1.55)	(1.00–1.50)	
3.07	**CMR**	—	—	0.38	—
(1.46–6.45)				(0.18–0.78)	
2.24	0.73	**Exercise ECG**	—	0.42	1.93
(1.22–4.11)	(0.28–1.88)			(0.14–1.30)	(1.39–2.67)
1.27	0.42	0.57	**SPECT-MPI**	0.87	—
(1.01–1.60)	(0.20–0.87)	(0.30–1.07)		(0.71–1.06)	
1.17	0.38	0.52	0.92	**Standard care**	2.95
(0.97–1.40)	(0.18–0.78)	(0.28–0.96)	(0.76–1.10)		(0.97–8.98)
4.31	1.40	1.93	3.38	3.69	**Stress echo**
(2.23–8.32)	(0.53–3.74)	(1.39–2.66)	(1.71–6.68)	(1.90–7.17)	

Odds ratios and their 95% confidence intervals are presented for referrals for invasive coronary angiography. Odds ratios in the lower triangle less than 1 favour the strategy in the column; odds ratios in the upper triangle less than 1 favour the strategy in the row. Cells with a dash indicate that no direct studies examined that particular comparison.

CCTA, coronary computed tomography angiography; CMR, cardiovascular magnetic resonance; ECG, electrocardiogram; echo, echocardiography; SPECT-MPI, single photon emission computed tomography–myocardial perfusion imaging.

**Box 2. The CINeMA web application.**

The CINeMA framework has been implemented in a freely available, user-friendly web application aiming to facilitate the evaluation of confidence in the results from network meta-analysis [13]. The web application is programmed in JavaScript, uses Docker, and is linked with R; in particular, packages *meta* and *netmeta* are used, and an R package to calculate the contribution of studies in network meta-analysis treatment effects [14,15]. Knowledge of these languages and technologies is not required to use CINeMA. The source code is available at https://github.com/esm-ispm-unibe-ch/cinema.

**Loading the data**

In the ‘My Projects’ tab, CINeMA users are able to upload a comma-separated values (csv) file with the by-treatment outcome study data and study-level risk of bias and indirectness judgments. The CINeMA web application can handle all the formats used in network meta-analysis (long or wide format, binary or continuous, arm-level or study-level data) and provides flexibility in labelling variables as desired by the user. A demo dataset is available under the ‘My Projects’ tab.

**Evaluating the confidence in the results from network meta-analysis**

A preview of the evidence (network plot and outcome data) and options concerning the analysis (fixed or random effects, effect measure, etc.) are available under the ‘Configuration’ tab. The next 6 tabs guide users to make informed assessments of the quality of evidence based on within-study bias, reporting bias, indirectness, imprecision, heterogeneity, and incoherence. Features include the percentage contribution matrix, relative treatment effects for each comparison, estimation of the heterogeneity variance, prediction intervals, and tests for the evaluation of the assumption of coherence.

**Summarising judgments**

The ‘Report’ tab includes a summary of the evaluations made in the 6 domains and gives users the possibility to either not downgrade, or downgrade by 1 or 2 levels each relative treatment effect. Users can download a report with the summary of their evaluations along with their final judgments. CINeMA is accompanied by documentation describing each step in detail (‘Documentation’ tab).

## Methodology

The framework was initially developed by a Cochrane Methods Group (Comparing Multiple Interventions Methods Group; https://methods.cochrane.org/cmi/about-us), based on previous work [4,16,17]. A preliminary version of the framework was published [5], and recent advances were subsequently implemented in a freely available web application [13]. Here we present the latest, refined version of the framework, which was developed in regular meetings between the authors, based on feedback by users of the CINeMA web application and participants at several workshops, including Cochrane webinars and workshops at Cochrane Colloquia (Cape Town, 2017; Edinburgh, 2018) and workshops at the World Health Organization (November 2017) and the National Institute for Health and Care Excellence (NICE) (February 2018).

### Within-study bias

#### Background and definitions

Within-study bias refers to shortcomings in the design or conduct of a study that can lead to an estimated relative treatment effect that systematically differs from the truth. In our framework we assume that studies have been assessed for risk of bias. The majority of published systematic reviews of randomised controlled trials currently use a tool developed by Cochrane to evaluate risk of bias [18]. This tool classifies studies as having low, unclear, or high risk of bias for various domains (such as allocation concealment, attrition, and blinding), with judgments summarised across domains. A recent revision of the tool takes a similar approach [19].

#### The CINeMA approach

While it is straightforward to gauge the impact of within-study biases in pairwise meta-analysis [20], this is more complex in network meta-analysis. The treatment comparison of interest might not have been tested directly in any trial, or tested in only a few small trials at high risk of bias. Thus, even when direct evidence is present, judgments about the relative treatment effect cannot ignore the risk of bias in the studies providing indirect evidence. In complex networks, indirect evidence is often obtained via several routes, including 1-step loops and loops involving several steps. It may then be insufficient to consider only the risk of bias in studies in a single prominent 1-step loop, as has previously been advocated [4,21]. This is because most studies in a network contribute more when their results are precise (e.g., large studies), when they provide direct evidence, or when the indirect evidence does not involve many steps. For example, studies in a 1-step indirect comparison contribute more than studies of the same precision in a 2-step indirect comparison. We can quantify the contribution made by each study to each relative treatment effect and present contributions in a percentage contribution matrix [22].

CINeMA combines the studies’ contributions with the risk of bias judgments to evaluate within-study bias for each estimate from a network meta-analysis. It uses the percentage contribution matrix to approximate the contribution of each study and then computes the percentage contribution from studies judged to be at low, moderate, and high risk of bias. Using different colours, study limitations in direct comparisons can be shown graphically in the network plot, while study limitations in the estimates from a network meta-analysis are presented for each comparison in bar charts.

It can be useful to assign judgments of ‘no concerns’, ‘some concerns’, or ‘major concerns’ about within-study bias according to the relative contributions from studies at high or moderate risk of bias. The contributions defining different levels of concern should be informed by sensitivity analysis. If the summary estimates from studies at high/moderate risk of bias are similar to those obtained from studies at low risk of bias, then even a large contribution from studies at high risk of bias will raise few concerns. The sensitivity analyses should be pre-specified in the study protocol to avoid data-driven conclusions.

#### Example: Comparing diagnostic strategies to detect coronary artery disease

Consider the comparison of exercise ECG versus standard care in the network meta-analysis described in Box 1, Fig 1A, and S1 Data. The direct evidence from a single study is at low risk of bias (study 12), so there is no within-study bias when interpreting the direct odds ratio of 0.42. However, indirect information from 7 studies that compare standard care and CCTA, from 1 study comparing exercise ECG and CCTA, and from 3 studies of stress echo contribute additional information to the odds ratio of 0.52 from the network meta-analysis. The risk of bias in the 11 studies providing indirect evidence varies (Fig 1A). Every study in the 2 1-step loops contributes information proportional to its precision (the inverse of the squared standard error, largely driven by sample size). Consequently, the within-study bias of the indirect evidence can be judged by considering that there is much information both from studies at high risk of bias (2,162 participants randomised) and from studies at low risk of bias (2,355 participants) and relatively little information from studies at moderate risk of bias (60 participants). Direct evidence from study number 12 (based on 130 participants), at low risk of bias, is considered separately, as it has greater influence than the indirect evidence.

Studies in the indirect comparisons contribute information not only proportional to their study precision but also according to their location in the network. Indirect evidence about exercise ECG versus SPECT-MPI comes from 2 1-step loops (via CCTA or via standard care) and 3 2-step loops (via CCTA–standard care, stress echo–standard care, standard care–CCTA) (Fig 1A). In each loop of evidence, a different subgroup of studies contributes indirect information, and the studies’ size and risk of bias vary. For the odds ratio from the network meta-analysis comparing exercise ECG and SPECT-MPI, study 2 (with sample size 400) is more influential than study 8 (with sample size 1,392) because study 2 contributes 1-step indirect evidence (via standard care) whereas study 8 contributes via 2 steps.

**Fig 2:**
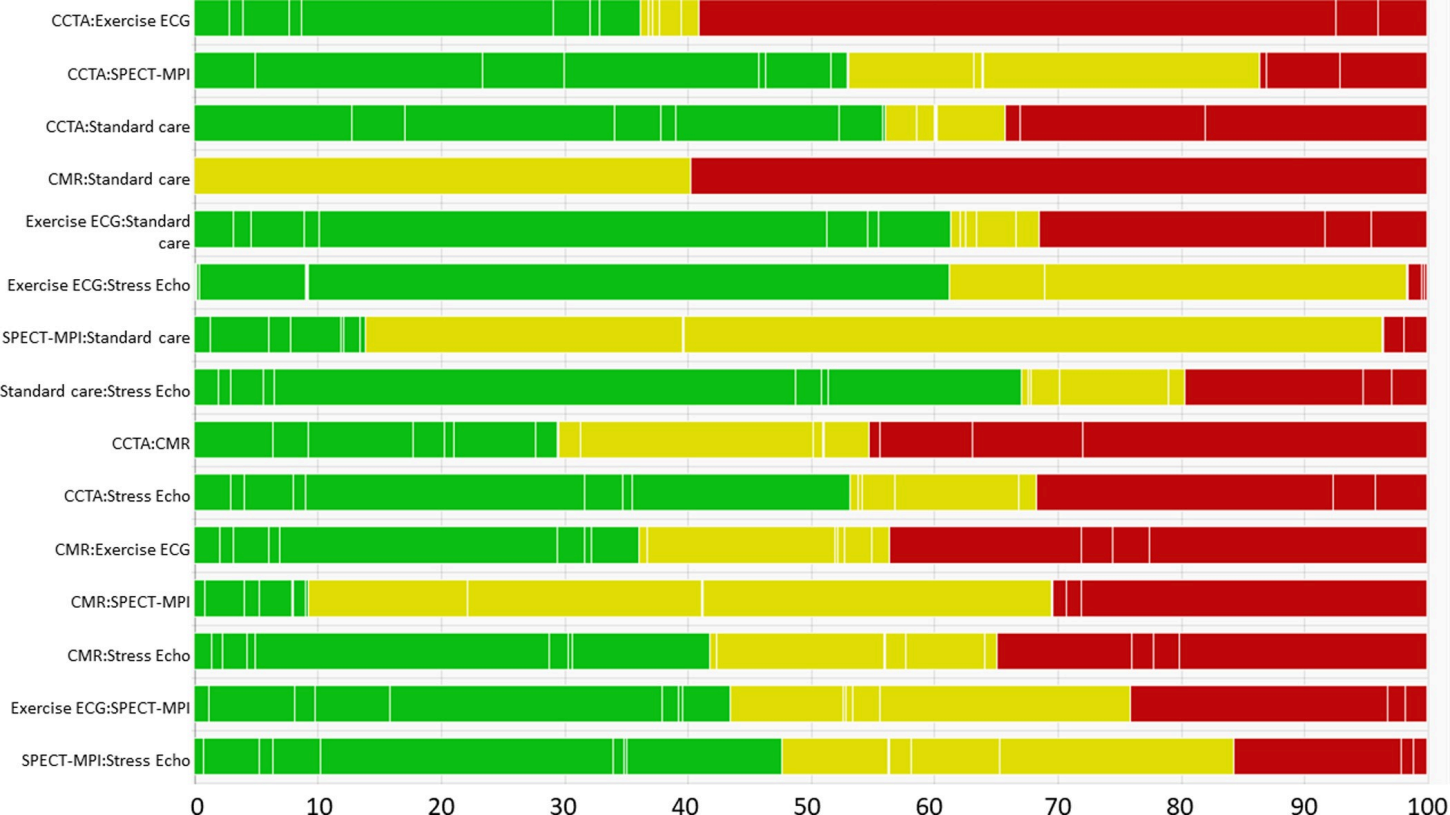
Risk of bias bar chart for the comparison of non-invasive diagnostic strategies for the detection of coronary artery disease. Each bar represents a relative treatment effect estimated from the network shown in Fig 1A. White vertical lines separate the percentage contribution of different studies. Each bar shows the percentage contribution from studies judged to be at low (green), moderate (yellow), and high (red) risk of bias.

Table 2 shows the percentage contribution matrix for the network, with columns representing studies grouped by comparison. The rows represent all relative treatment effects from network meta-analysis. The matrix entries show how much each study contributes to the estimation of each relative treatment effect. Combined with the risk of bias judgments, this information can be presented as a bar chart, as shown in Fig 2. The larger the contribution from studies at high or moderate risk of bias, the greater the concern about study limitations. For example, studies at low risk bias contribute 44% to the estimation of the indirect evidence for the comparison of exercise ECG with SPECT-MPI, with the contributions from studies with moderate and high risk of bias being 32% and 24%, respectively.

**Table 2:** The percentage contribution matrix for the network presented in Fig 1A.

Network meta-analysis comparison	Direct comparison (number of studies)																			
	CCTA versus exercise ECG (1)	CCTA versus SPECT-MPI (2)		CCTA versus standard care (7)							CMR versus standard care (2)		Exercise ECG versus standard care (1)	Exercise ECG versus stress echo (4)				SPECT-MPI versus standard care (2)		Standard care versus stress echo (1)
	Study 3	Study 2	Study 9	Study 1	Study 10	Study 13	Study 14	Study 4	Study 7	Study 8	Study 11	Study 6	Study 12	Study 12	Study 15	Study 16	Study 17	Study 18	Study 5	Study 12
***Mixed estimates***																				
CCTA:exercise ECG	52	1	1	3	0	3	1	3	4	4	0	0	14	0	3	0	2	1	1	6
CCTA:SPECT-MPI	1	18	16	5	1	5	1	6	7	7	0	0	0	0	0	0	0	22	10	0
CCTA:standard care	1	4	4	13	2	13	3	15	18	17	0	0	1	0	0	0	0	6	3	0
CMR:standard care	0	0	0	0	0	0	0	0	0	0	60	40	0	0	0	0	0	0	0	0
Exercise ECG:standard care	23	1	1	3	0	3	1	4	5	4	0	0	30	1	6	1	3	2	1	11
Exercise ECG:stress echo	1	0	0	0	0	0	0	0	0	0	0	0	1	5	52	8	29	0	0	2
SPECT-MPI:standard care	0	5	4	1	0	1	0	2	2	2	0	0	0	0	0	0	0	57	26	0
Standard care:stress echo	14	1	1	2	0	2	1	2	3	3	0	0	14	2	16	2	9	1	1	27
***Indirect estimates***																				
CCTA:CMR	1	3	2	6	1	7	2	8	9	8	28	19	1	0	0	0	0	4	2	0
CCTA:stress echo	24	1	1	3	0	3	1	3	4	4	0	0	8	2	18	3	10	1	1	13
CMR:exercise ECG	16	1	1	2	0	2	1	3	3	3	22	15	15	0	4	1	2	1	1	7
CMR:SPECT-MPI	0	3	3	1	0	1	0	1	1	1	28	19	0	0	0	0	0	28	13	0
CMR:stress echo	11	1	1	1	0	2	0	2	2	2	20	14	9	1	11	2	6	1	0	13
Exercise ECG:SPECT-MPI	21	7	6	1	0	1	0	1	2	2	0	0	15	0	4	1	2	20	9	7
SPECT-MPI:stress echo	14	5	4	1	0	1	0	1	1	1	0	0	9	1	13	2	7	19	9	13

The columns refer to the studies (grouped by comparison), and the rows refer to the relative treatment effects (grouped into mixed and indirect estimates) from network meta-analysis. The entries show how much each study contributes (as percentage) to the estimation of the relative treatment effect. Mixed relative treatment effects are estimated using both direct and indirect evidence.

CCTA, coronary computed tomography angiography; CMR, cardiovascular magnetic resonance; ECG, electrocardiogram; echo, echocardiography; SPECT-MPI, single photon emission computed tomography–myocardial perfusion imaging.

CCTA, coronary computed tomography angiography; CMR, cardiovascular magnetic resonance; ECG, electrocardiogram; echo, echocardiography; SPECT-MPI, single photon emission computed tomography–myocardial perfusion imaging.

The CINeMA software facilitates judgments, based on the data presented in the bar graphs combined with specific rules. For example, a weighted average of the risk of bias can be computed by assigning scores of −1, 0, and 1 to low, moderate, and high risk of bias, respectively. For the comparison exercise ECG versus SPECT-MPI, this would produce a weighted score of 0.44 × −1 + 0.32 × 0 + 0.24 × 1 = −0.20, which corresponds to ‘some concerns’.

### Reporting bias

#### Background and definitions

Reporting bias occurs when the results included in the systematic review are not a representative sample of the results generated by studies undertaken. This phenomenon can be the result of the suppression of statistically non-significant (or ‘negative’) findings (publication bias), their delayed publication (time-lag bias), or omission of unfavourable study results from study reports (outcome reporting bias). The presence and the impact of such biases has been well documented [23–30]. Reporting bias is a missing data problem, and hence it is difficult to conclude with certainty for or against its presence in a given dataset. Consequently, and in agreement with the GRADE system, CINeMA assumes 2 possible descriptions for reporting bias: suspected and undetected.

#### The CINeMA approach

Conditions associated with suspected reporting bias include the following:

- There is a failure to include unpublished data and data from grey literature.
- The meta-analysis is based on a small number of positive early findings, for example for a drug newly introduced on the market (as early evidence is likely to overestimate its efficacy and safety) [31].
- The treatment comparison is studied exclusively or primarily in industry-funded trials [32,33].
- There is previous evidence documenting the presence of reporting bias; for example, the study by Turner et al. documented publication bias in placebo-controlled antidepressant trials [34].

Reporting bias is considered undetected under the following conditions:

- Data from unpublished studies have been identified, and their findings agree with those in published studies.
- There is a tradition of prospective trial registration in the field, and protocols or clinical trial registries do not indicate important discrepancies with published reports.
- Empirical examination of patterns of results between small and large studies, using comparison-adjusted [35,36], regression [37], or selection [38] models, do not indicate that results from small studies differ from those in published studies.

See S1 Text for a worked example based on the antidepressants network.

### Indirectness

#### Background and definitions

Systematic reviews are based on a focused research question, with a clearly defined population, intervention, and setting of interest. In the GRADE framework for pairwise meta-analysis, indirectness refers to the relevance of the included studies to the research question [39]. Study populations, interventions, outcomes, and study settings might not be representative of the settings, populations, or outcomes about which reviewers want to make inferences. For example, a systematic review about treating the general adult population might identify studies only in elderly men; these studies will have an indirect relevance [40].

#### The CINeMA approach

Each study included in the network is evaluated according to its relevance to the research question, classified into low, moderate, or high indirectness. Note that only participant, intervention, and outcome characteristics that are likely associated with the relative effect of an intervention (i.e., effect-modifying variables) should be considered. Then, the study-level judgments can be combined with the percentage contribution matrix to produce a bar chart similar to the one presented in Fig 2, and the contribution from studies of high or moderate indirectness assessed.

Indirectness addresses the issue of transitivity in network meta-analysis. Transitivity means that we can learn about the relative treatment effect of treatment A versus treatment B from an indirect comparison via treatment C. This holds when the distributions of all effect modifiers are comparable in studies of A versus C and studies of B versus C. Different distributions of effect modifiers across comparisons indicate intransitivity. Evaluation of the distribution of effect modifiers is only possible when enough studies are available per comparison. Also, assessment of transitivity will be challenging or impossible for interventions that are poorly connected to the network. A further potential obstacle is that details of important effect modifiers might not be reported. For these reasons, we recommend that the network structure and the amount of available data are considered, and that judgments are on the side of caution, as highlighted in the worked example for the antidepressants network provided in S1 Text.

### Imprecision

#### Background and definitions

A key advantage of network meta-analysis compared with pairwise meta-analysis is the gain in precision [41] by adding indirect evidence to direct evidence. To evaluate imprecision the relative treatment effect that represents a clinically important difference needs to be defined. At its simplest, this treatment effect corresponds to any beneficial effect. In this case even a small difference is considered important, leading to one treatment being preferred over another. Alternatively, ranges may be defined that divide relative treatment effects into 3 categories: ‘favours X’, ‘no important difference between X and Y’, and ‘favours Y’. The middle range is the ‘range of equivalence’, which corresponds to clinically unimportant differences between interventions. The range of equivalence should be defined with reference to absolute risk differences that matter to patients. The range of equivalence can be symmetrical (when a clinically important benefit is the reciprocal of a clinically important harm, on a relative scale) or asymmetrical (when clinically important differences vary by direction of effect). For simplicity, we will assume symmetrical ranges of equivalence.

#### The CINeMA approach

**Fig 3:**
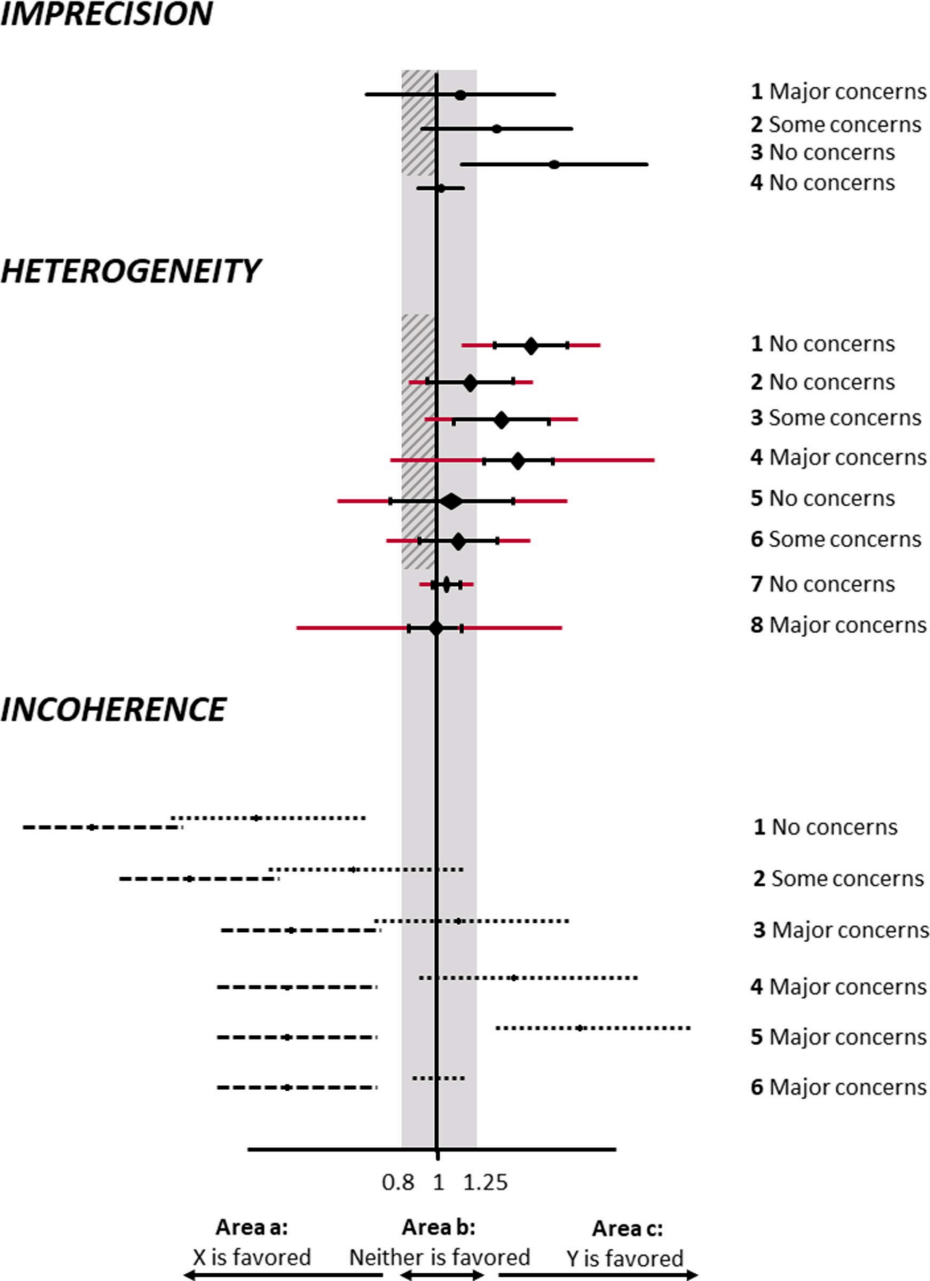
CINeMA rules to assess imprecision, heterogeneity, and incoherence of network treatment effects. Black lines show confidence intervals, and red lines prediction intervals. The shaded grey area represents values that favour neither of the competing interventions: the range of equivalence, from 0.8 to 1.25. The hatched area shows the interval between the no effect line and a clinically important effect in the opposite direction to the observed effect. For incoherence, dashed lines represent direct effects, and dotted lines indirect effects.

CINeMA compares the treatment effects included in the 95% confidence interval with the range of equivalence, as illustrated in the upper part of Fig 3. A rating of ‘major concerns’ is assigned to a treatment effect if the 95% confidence interval extends beyond the area of equivalence on the opposite side of the no effect line as the point estimate, so that the estimated treatment effect is compatible with clinically important effects in both directions (imprecision scenario 1 in Fig 3). A rating of ‘some concerns’ is assigned if the confidence interval extends into but not beyond the area of equivalence on the opposite side of the no effect line (imprecision scenario 2 in Fig 3). There are ‘no concerns’ if the confidence interval is entirely on one side of the no effect line (imprecision scenario 3 in Fig 3), or if it is entirely within the area of equivalence (imprecision scenario 4).

#### Example: Adverse events of statins

**Fig 4:**
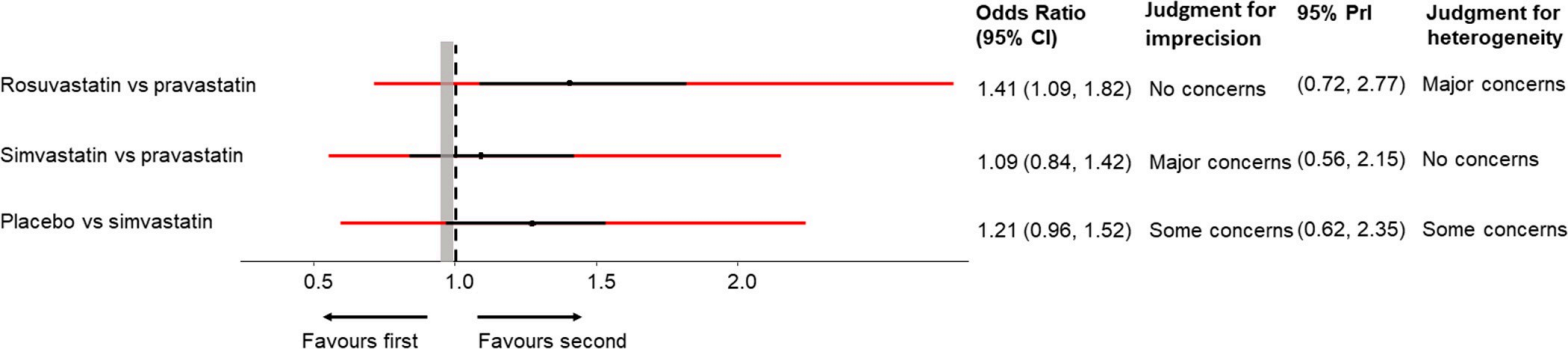
Odds ratios for treatment discontinuation due to adverse effects from the network of statins with their 95% CIs and their 95% PrIs. Black lines represent 95% CIs, and red lines represent 95% PrIs. The range of equivalence is from 0.95 to 1.05. CI, confidence interval; PrI, prediction interval; vs, versus.

Consider the network comparing adverse events of different statins, introduced in Box 1 and shown in Fig 1B [11]. Let us assume a range of equivalence that translates into an odds ratio greater than 1.05 or below 0.95 ($=\frac{1}{1.05}$) representing a clinically important effect. For odds ratios greater than 1, we compare the 95% confidence interval with the opposite half of the range of equivalence (0.95, 1). The 95% confidence interval of rosuvastatin versus pravastatin is quite wide (1.09 to 1.82; Fig 4), but any treatment effect in this range would lead to the conclusion that pravastatin is safer than rosuvastatin. Thus, imprecision does not reduce the confidence that can be placed in the comparison of pravastatin with rosuvastatin (‘no concerns’). The 95% confidence interval of simvastatin versus pravastatin is wider (0.84 to 1.42) and covers all 3 areas, extending below 0.95, thus favouring pravastatin, and above 1, thus favouring simvastatin, and including no important difference. ‘Major concerns’ on imprecision therefore applies. The comparison of placebo versus simvastatin is more certain, but it is unclear which drug has fewer adverse effects: most estimates within the 95% confidence interval favour simvastatin, but the interval crosses into the (0.95, 1) range. A rating of ‘some concerns’ is appropriate.

### Heterogeneity

#### Background and definitions

Variability in the results of studies influences our confidence in the point estimate of a relative treatment effect. If this variability reflects genuine differences between studies, rather than random variation, it is called heterogeneity. GRADE uses the term inconsistency [42] rather than heterogeneity. In network meta-analysis, there may be variation in treatment effects between studies, i.e., heterogeneity, but also variation between direct and indirect sources of evidence. The latter is called incoherence [43–46] and is discussed below. The 2 concepts are related; incoherence can be seen as a special form of heterogeneity.

There are several ways of measuring heterogeneity in a set of trials. The variance of the distribution of the underlying treatment effects (τ^2^) is a measure of the magnitude of heterogeneity. This variance can be estimated for each pairwise meta-analysis, and, under the assumption of a single variance across comparisons, a common heterogeneity variance can be obtained for the whole network. The magnitude of τ^2^ is usefully expressed as a prediction interval, which shows where the true effect of a new study similar to the existing studies is expected to lie [47].

#### The CINeMA approach

As for imprecision, the CINeMA approach to heterogeneity involves comparisons of results with the pre-specified range of clinical equivalence. Specifically, we examine both the confidence intervals (which do not capture heterogeneity) and prediction intervals (which do capture heterogeneity) in relation to the range of equivalence. Heterogeneity is important if a prediction interval includes values that lead to a different conclusion than an assessment based on the confidence interval. The middle part of Fig 3 illustrates the CINeMA rules for a range of equivalence of 0.8 to 1.25. When confidence and prediction intervals lead to the same conclusions (heterogeneity scenarios 1, 2, 5, and 7 in the middle part of Fig 3), then there are ‘no concerns’ about heterogeneity. When the confidence intervals and the prediction intervals lead to conclusions that are somewhat different but of lesser impact for decision-making, CINeMA concludes that there are ‘some concerns’ about heterogeneity (heterogeneity scenarios 3 and 6 in Fig 3).

If there are very few trials, the amount of heterogeneity is poorly estimated and prediction intervals are unreliable. Turner et al. and Rhodes et al. analysed many meta-analyses of binary and continuous outcomes, categorised them according to the outcome and type of intervention and comparison, and derived empirical distributions of heterogeneity values [48,49]. These empirical distributions can help to interpret the magnitude of heterogeneity, complementing considerations based on prediction intervals.

#### Example: Adverse events of statins

In the statins example, we assumed a range of equivalence of 0.95 to 1.05. The prediction interval for simvastatin versus pravastatin is wide (Fig 4). However, the confidence interval for this comparison already extended to both sides of the (0.95, 1) range; thus, the heterogeneity does not change the conclusion. The confidence interval for rosuvastatin versus pravastatin lies entirely above the (0.95, 1) range, and there are ‘no concerns’ regarding imprecision. However, the prediction interval crosses both boundaries (0.95 and 1), and we therefore have ‘major concerns’ about the impact of heterogeneity. Similar considerations result in ‘some concerns’ regarding heterogeneity for the comparison of placebo versus simvastatin.

### Incoherence

#### Background and definitions

Transitivity stipulates that we can compare 2 treatments indirectly via an intermediate treatment node. Incoherence is the statistical manifestation of intransitivity; if transitivity holds, the direct and indirect evidence should be in agreement and coherent [50,51]. Conversely, if estimates from direct and indirect evidence disagree, we conclude that transitivity does not hold and that there is incoherence.

To quantify incoherence the agreement between direct and indirect evidence can be examined for specific comparisons in the network, often referred to as a local approach. For example, SIDE or ‘node splitting’ [43] compares the direct and indirect evidence for each comparison to estimate an inconsistency factor with a confidence interval. The inconsistency factor is calculated as the difference or ratio of the 2 estimates. This method can only be applied to comparisons that are informed by both direct and indirect evidence. Consider the incoherence scenario 1 in the lower part of Fig 3: The studies directly comparing the 2 treatments result in a direct odds ratio of 0.48 (0.42 to 0.54), while the studies that provide indirect evidence produce an odds ratio of 0.61 (0.52 to 0.70). The inconsistency factor in this example (the ratio of indirect to direct odds ratio) is 1.28, with a confidence interval of 1.05 to 1.55 and a *p*-value of 0.07. A simpler version of SIDE considers a single loop in the network (loop-specific approach [52]).

The global approach is an alternative approach to quantifying incoherence. It models all treatment effects and all possible inconsistency factors simultaneously, resulting in an ‘omnibus test’ of incoherence in the whole network. The design-by-treatment interaction test is such a global test [45,46]. An overview of other methods for testing incoherence can be found elsewhere [44,53].

#### The CINeMA approach

Both global and local incoherence tests have low power [54,55], and it is therefore important to consider the inconsistency factors as well as their uncertainty. A large inconsistency factor may indicate a biased direct or indirect estimate. As for imprecision and heterogeneity, the CINeMA approach to incoherence considers the impact on clinical implications, based on visual inspection of the 95% confidence intervals of direct and indirect odds ratios and the range of equivalence. The rules implemented in CINeMA are illustrated in the lower part of Fig 3. The inconsistency factor using the SIDE approach is the same for the first 3 incoherence scenarios in Fig 3 (ratio of odds ratios 1.28 with confidence interval 1.05 to 1.55), but their position relative to the range of equivalence differs and affects the interpretation of incoherence. In incoherence scenario 1, the 95% confidence intervals for both direct and indirect odds ratios lie below the range of equivalence: treatment X is clearly favoured, and there are ‘no concerns’ regarding incoherence. In incoherence scenario 2, the 95% confidence interval of the indirect odds ratio straddles the range of equivalence, while for the direct odds ratio the 95% confidence interval lies entirely below 0.8. In this situation, a judgment of ‘some concerns’ is appropriate. In incoherence scenario 3, the 95% confidence intervals of the odds ratios from direct and indirect comparisons share only the area below 0.8, whereas only the 95% confidence interval of the indirect odds ratio lies within and above the range of equivalence. Therefore, the disagreement leads to a rating of ‘major concerns’.

Note that in the scenarios described above, the *p*-value from SIDE is 0.07. As a general rule, there are ‘no concerns’ if the *p*-value is greater than 0.10, independent of the position of the 95% confidence intervals with respect to the range of equivalence, because the evidence for incoherence is weak (*p* > 0.10). This rule can be overwritten on a case by case basis.

Incoherence can only be evaluated using local approaches when both direct and indirect estimates are available for the comparison, as was the case for the incoherence scenarios in Fig 3. If there is only direct or only indirect evidence, we can neither estimate an inconsistency factor nor judge potential implications with respect to the range of equivalence. In this situation, CINeMA considers that there are ‘major concerns’ if the *p*-value of the global design-by-treatment interaction test is <0.05, ‘some concerns’ if it is between 0.05 and 0.10, and ‘no concerns’ if it is >0.10. There are also ‘major concerns’ if the design-by-treatment interaction test statistic cannot be computed due to the absence of closed loops in the network.

In S1 Text we provide a worked example for the antidepressants network.

### Summarising judgments across the 6 domains

Τhe final output of CINeMA is a table with the level of concern for each of the 6 domains. Reviewers may then choose to summarise judgments across domains using the 4 levels of confidence of the GRADE approach: very low, low, moderate, or high [4]. For this purpose, one may start at high confidence and drop the level of confidence by 1 step for each domain with some concerns, and by 2 levels for each domain with major concerns. However, it is important to note that domains are interconnected: factors that may reduce the confidence in a treatment effect may affect more than 1 domain. Indirectness includes considerations on intransitivity, which manifests itself in the data as statistical incoherence. Heterogeneity will increase imprecision in treatment effects and may be related to variability in within-study bias or the presence of reporting bias. Furthermore, in the presence of heterogeneity, the ability to detect important incoherence will decrease [54]. The 6 CINeMA domains should therefore be considered jointly rather than in isolation, avoiding downgrading the overall level of confidence more than once for related concerns. For example, for the citalopram versus venlafaxine comparison in the antidepressants example, we have ‘some concerns’ for imprecision and heterogeneity and ‘major concerns’ for incoherence (see S1 Text). However, downgrading by 2 levels is sufficient in this situation, because imprecision, heterogeneity, and incoherence are interconnected.

## Discussion

We have outlined and illustrated the CINeMA approach for evaluating confidence in treatment effect estimates from network meta-analysis, covering the 6 domains of within-study bias, reporting bias, indirectness, imprecision, heterogeneity, and incoherence. We differentiate between the 3 sources of variability in a network, namely, imprecision, heterogeneity, and incoherence, and we consider the impact that each source might have on decisions for treatment. Our approach avoids the selective use of indirect evidence, while considering the characteristics of all studies included in the network. In other words, we are not using assessments of confidence to decide whether to present direct or indirect (or combined) evidence, as has been suggested by others [4,5]. The web application greatly facilitates the implementation of all steps involved in the application of CINeMA [13] and makes the approach easy to implement even for very large networks. Researchers should not, however, naively rely on the software’s programmed rules. The CINeMA application safeguards against transcription errors and thus will enhance reproducibility, but judgments should be revisited and reconsidered, taking into account the particularities of each network meta-analysis. This paper and the CINeMA tool extend the framework previously described by Salanti and colleagues [5]. Our original framework also addressed the credibility of a treatment hierarchy, which we plan to develop further and implement in CINeMA in the future.

Any evaluation of the confidence in evidence synthesis results will inevitably involve some subjectivity. Our approach is no exception. While the use of bar charts to gauge the impact of within-study bias and indirectness provides a consistent assessment across all comparisons in the network, their summary is difficult. Defining the range of equivalence will often be subjective, and might be influenced by the data. Further limitations of the framework are associated with the fact that published articles are used to make judgments, and these reports do not necessarily reflect the way studies were undertaken. For instance, judging indirectness requires study data to be collected on pre-specified effect modifiers, and incomplete reporting will inevitably impact on the reliability of judgments.

A consequence of the inherent subjectivity of the system is that interrater agreement may be modest. Studies of the reproducibility of assessments made by researchers using CINeMA will be required in this context. However, we believe that transparency is key: although in CINeMA judgments may differ across reviewers, they are made using explicit criteria. These should be specified in the review protocol so that data-driven decisions are avoided.

A GRADE working group developed [4] and subsequently refined [21] an approach for rating the quality of treatment effect estimates from network meta-analysis. There are many similarities but also some notable differences between CINeMA and the GRADE approach. For example, Puhan et al. [4] suggest a process of deciding whether indirect estimates are of sufficient certainty to combine them with the direct estimates. In contrast, CINeMA evaluates relative treatment effects without considering separately the direct and indirect sources. Evaluation of the impact of within-study bias also differs substantially between the 2 approaches. The GRADE approach considers within-study bias of the most influential 1-step loop for each treatment effect [4,21], which discards a large amount of information and makes the approach difficult to apply to large networks. We believe that the CINeMA approach, which is based on the percentage contribution matrix and considers the impact of every study included in the network, is preferable. In contrast to the GRADE approach, CINeMA does not rely on metrics for judging heterogeneity and incoherence. Instead it considers the likely impact of these issues on clinical decisions. Yet another approach to assessing the credibility of conclusions from network meta-analysis explores how robust treatment recommendations are to potential degrees of bias in the evidence [56]. The method is easy to apply but exclusively focuses on the impact of bias and does not explicitly address heterogeneity, indirectness, and incoherence.

Evidence synthesis is increasingly used by national and international medical societies and agencies [57,58] to inform decisions about the clinical effectiveness and cost-effectiveness of medical interventions, by clinical guideline panels to recommend one drug over another, and by clinicians to prescribe a treatment or recommend a diagnostic procedure for individual patients. However, published network meta-analyses seldom evaluate confidence in relative treatment effects [1]. Through the free, open-source CINeMA software (see Box 2), our approach can be routinely applied to any network meta-analysis [13,59]. The suggested framework operationalises, simplifies, and accelerates the process of evaluation of results from large and complex networks without compromising statistical and methodological rigor. In conclusion, we believe the CINeMA framework is a transparent, rigorous, and comprehensive system for evaluating the confidence of treatment effect estimates from network meta-analysis.

## Supporting information

S1 Data Data from a network of randomised controlled trials comparing non-invasive diagnostic strategies for the detection of coronary artery disease in patients presenting with symptoms suggestive of acute coronary syndrome. The data were originally published by Siontis et al. [10].
(DOCX)

S2 Data Data from a network of randomised controlled trials comparing adverse effects of statins. The data were originally published by Naci et al. [11]. id, ID of the study; n, sample size; r, number of adverse effects; t, treatment name.
(DOCX)

S1 Table Network meta-analysis results from the network of randomised controlled trials comparing adverse effects of statins. Odds ratios and their 95% confidence intervals are presented. The odds ratios presented in the upper triangle are the reciprocals of the odds ratios presented in the lower triangle. Odds ratios less than 1 favour the treatment specified in the row.
(DOCX)

S2 Table Results from SIDE for 3 network comparisons of the network of statins. OR, odds ratio; SIDE, Separating Indirect from Direct Evidence.
(DOCX)

S1 Text Worked example: Antidepressants for moderate and major depression. (DOCX)

## Abbreviations

CCTA: coronary computed tomography angiography
CINeMA: Confidence in Network Meta-Analysis
CMR: cardiovascular magnetic resonance
ECG: electrocardiogram, echo, echocardiography
GRADE: Grading of Recommendations Assessment, Development and Evaluation
SIDE: Separating Indirect from Direct Evidence
SPECT-MPI: single photon emission computed tomography–myocardial perfusion imaging
