# Supplementary material for: CINeMA: An approach for assessing confidence in the results of a network meta-analysis
Source: PLoS Med. 2020 Apr 3;17(4):e1003082. doi: 10.1371/journal.pmed.1003082 (PMC7122720; doi:10.1371/journal.pmed.1003082)
Supplement: S1 Data — The data were originally published by Siontis et al. [10]. (DOCX) [file pmed.1003082.s001.docx]

| id | trial | group | n | r | rob | t |
| --- | --- | --- | --- | --- | --- | --- |
| 1 | BEACONR1 | Anatomical testing | 250 | 41 | 1 | CCTA |
| 1 | BEACONR1 | Standard care | 250 | 31 | 1 | Standard care |
| 2 | Levsky JM., et al.R2 | Anatomical testing | 200 | 30 | 1 | CCTA |
| 2 | Levsky JM., et al.R2 | Functional testing | 200 | 32 | 1 | SPECT-MPI |
| 3 | CT-COMPARER3 | Anatomical testing | 322 | 26 | 3 | CCTA |
| 3 | CT-COMPARER3 | Functional testing | 240 | 9 | 3 | Exercise ECG |
| 4 | CATCHR4,R5 | Anatomical testing | 299 | 49 | 3 | CCTA |
| 4 | CATCHR4,R5 | Standard care | 301 | 36 | 3 | Standard care |
| 5 | Lim SH., et al.R6 | Functional testing | 1126 | 73 | 2 | SPECT-MPI |
| 5 | Lim SH., et al.R6 | Standard care | 564 | 56 | 2 | Standard care |
| 6 | Miller CD., et al.R7 | CMR | 52 | 5 | 2 | CMR |
| 6 | Miller CD., et al.R7 | Standard care | 53 | 11 | 2 | Standard care |
| 7 | ROMICAT-IIR8 | Anatomical testing | 501 | 59 | 3 | CCTA |
| 7 | ROMICAT-IIR8 | Standard care | 499 | 40 | 3 | Standard care |
| 8 | ACRIN-PAR9,R10 | Anatomical testing | 929 | 69 | 1 | CCTA |
| 8 | ACRIN-PAR9,R10 | Standard care | 463 | 32 | 1 | Standard care |
| 9 | CT-STATR11 | Anatomical testing | 375 | 26 | 1 | CCTA |
| 9 | CT-STATR11 | Functional testing | 374 | 22 | 1 | SPECT-MPI |
| 10 | Miller AH., et al.R12 | Anatomical testing | 30 | 4 | 2 | CCTA |
| 10 | Miller AH., et al.R12 | Standard care | 30 | 4 | 2 | Standard care |
| 11 | Miller CD., et al.R13,R14 | CMR | 52 | 8 | 3 | CMR |
| 11 | Miller CD., et al.R13,R14 | Standard care | 57 | 19 | 3 | Standard care |
| 12 | Nucifora G., et al.R15 | Functional testing | 77 | 5 | 1 | Stress Echo |
| 12 | Nucifora G., et al.R15 | Functional testing | 75 | 9 | 1 | Exercise ECG |
| 12 | Nucifora G., et al.R15 | Standard care | 55 | 8 | 1 | Standard care |
| 13 | Chang SA., et al.R16 | Anatomical testing | 133 | 47 | 1 | CCTA |
| 13 | Chang SA., et al.R16 | Standard care | 133 | 57 | 1 | Standard care |
| 14 | Goldstein JA., et al.R17 | Anatomical testing | 99 | 12 | 1 | CCTA |
| 14 | Goldstein JA., et al.R17 | Standard care | 98 | 7 | 1 | Standard care |
| 15 | Jeetley P., et al.R18 | Functional testing | 215 | 41 | 1 | Stress Echo |
| 15 | Jeetley P., et al.R18 | Functional testing | 218 | 72 | 1 | Exercise ECG |
| 16 | Nucifora G., et al.R19 | Functional testing | 110 | 6 | 2 | Stress Echo |
| 16 | Nucifora G., et al.R19 | Functional testing | 89 | 6 | 2 | Exercise ECG |
| 17 | Jeetley P., et al.R20 | Functional testing | 148 | 21 | 2 | Stress Echo |
| 17 | Jeetley P., et al.R20 | Functional testing | 154 | 36 | 2 | Exercise ECG |
| 18 | Udelson JE., et al.R21 | Functional testing | 1215 | 156 | 2 | SPECT-MPI |
| 18 | Udelson JE., et al.R21 | Standard care | 1260 | 162 | 2 | Standard care |
