# Supplementary material for: CINeMA: An approach for assessing confidence in the results of a network meta-analysis
Source: PLoS Med. 2020 Apr 3;17(4):e1003082. doi: 10.1371/journal.pmed.1003082 (PMC7122720; doi:10.1371/journal.pmed.1003082)
Supplement: S2 Data — The data were originally published by Naci et al. [11]. id, ID of the study; n, sample size; r, number of adverse effects; t, treatment name. (DOCX) [file pmed.1003082.s002.docx]

| year | study | id | t | r | n |
| --- | --- | --- | --- | --- | --- |
| 1993 | PMSG | 1 | pravastatin | 25 | 530 |
| 1993 | PMSG | 1 | placebo | 33 | 532 |
| 1993 | SPSG | 2 | simvastatin | 5 | 275 |
| 1993 | SPSG | 2 | pravastatin | 5 | 275 |
| 1993 | LPSG | 3 | lovastatin | 10 | 339 |
| 1993 | LPSG | 3 | pravastatin | 8 | 333 |
| 1993 | MARS | 4 | lovastatin | 3 | 123 |
| 1993 | MARS | 4 | placebo | 6 | 124 |
| 1994 | 4S | 5 | placebo | 129 | 2223 |
| 1994 | 4S | 5 | simvastatin | 126 | 2221 |
| 1994 | PMSG-Diabetes | 6 | pravastatin | 2 | 167 |
| 1994 | PMSG-Diabetes | 6 | placebo | 9 | 158 |
| 1994 | EXCEL | 7 | placebo | 100 | 1663 |
| 1994 | EXCEL | 7 | lovastatin | 329 | 6582 |
| 1994 | OCS | 8 | simvastatin | 18 | 414 |
| 1994 | OCS | 8 | placebo | 6 | 207 |
| 1995 | Jacobson | 9 | pravastatin | 9 | 182 |
| 1995 | Jacobson | 9 | placebo | 1 | 63 |
| 1995 | REGRESS | 10 | pravastatin | 16 | 450 |
| 1995 | REGRESS | 10 | placebo | 10 | 434 |
| 1995 | KAPS | 11 | placebo | 12 | 223 |
| 1995 | KAPS | 11 | pravastatin | 8 | 224 |
| 1995 | WOSCOPS | 12 | placebo | 106 | 3293 |
| 1995 | WOSCOPS | 12 | pravastatin | 116 | 3302 |
| 1995 | Guillen | 13 | placebo | 1 | 74 |
| 1995 | Guillen | 13 | pravastatin | 0 | 76 |
| 1996 | SHIGA Pravastatin study | 14 | pravastatin | 2 | 102 |
| 1996 | SHIGA Pravastatin study | 14 | placebo | 0 | 105 |
| 1996 | CARE | 15 | placebo | 74 | 2078 |
| 1996 | CARE | 15 | pravastatin | 45 | 2081 |
| 1996 | QLMG | 16 | lovastatin | 3 | 211 |
| 1996 | QLMG | 16 | pravastatin | 4 | 215 |
| 1996 | CHESS | 17 | simvastatin | 27 | 453 |
| 1996 | CHESS | 17 | atorvastatin | 65 | 464 |
| 1997 | Bertolini | 18 | atorvastatin | 7 | 227 |
| 1997 | Bertolini | 18 | pravastatin | 2 | 78 |
| 1997 | ASG-I | 19 | atorvastatin | 16 | 529 |
| 1997 | ASG-I | 19 | lovastatin | 5 | 120 |
| 1998 | AFCAPS-TexCAPS | 20 | placebo | 455 | 3301 |
| 1998 | AFCAPS-TexCAPS | 20 | lovastatin | 449 | 3304 |
| 1998 | Brown | 21 | atorvastatin | 3 | 78 |
| 1998 | Brown | 21 | fluvastatin | 4 | 76 |
| 1998 | Brown | 21 | lovastatin | 2 | 78 |
| 1998 | Brown | 21 | simvastatin | 2 | 76 |
| 1999 | TARGET TANGIBLE | 22 | atorvastatin | 89 | 1897 |
| 1999 | TARGET TANGIBLE | 22 | simvastatin | 45 | 959 |
| 1999 | Riegger | 23 | fluvastatin | 11 | 187 |
| 1999 | Riegger | 23 | placebo | 8 | 178 |
| 1999 | IQLMG | 24 | simvastatin | 7 | 194 |
| 1999 | IQLMG | 24 | pravastatin | 7 | 193 |
| 1999 | FLARE | 25 | fluvastatin | 4 | 409 |
| 1999 | FLARE | 25 | placebo | 11 | 425 |
| 2000 | Barter | 26 | atorvastatin | 48 | 691 |
| 2000 | Barter | 26 | simvastatin | 24 | 337 |
| 2000 | Farnier | 27 | atorvastatin | 1 | 109 |
| 2000 | Farnier | 27 | simvastatin | 1 | 163 |
| 2000 | Stein | 28 | placebo | 0 | 130 |
| 2000 | Stein | 28 | simvastatin | 1 | 260 |
| 2000 | Recto | 29 | simvastatin | 1 | 251 |
| 2000 | Recto | 29 | atorvastatin | 5 | 251 |
| 2000 | Gentile | 30 | atorvastatin | 1.5 | 85 |
| 2000 | Gentile | 30 | simvastatin | 0.5 | 79 |
| 2000 | Gentile | 30 | pravastatin | 1.5 | 82 |
| 2000 | Gentile | 30 | lovastatin | 1.5 | 81 |
| 2000 | Gentile | 30 | placebo | 0.5 | 87 |
| 2001 | ASSET | 31 | atorvastatin | 7 | 730 |
| 2001 | ASSET | 31 | simvastatin | 7 | 694 |
| 2001 | MIRACL | 32 | placebo | 33 | 1548 |
| 2001 | MIRACL | 32 | atorvastatin | 40 | 1538 |
| 2001 | Paoletti | 33 | rosuvastatin | 8 | 230 |
| 2001 | Paoletti | 33 | pravastatin | 3 | 136 |
| 2001 | Paoletti | 33 | simvastatin | 1 | 129 |
| 2001 | Andrews | 34 | atorvastatin | 129 | 1902 |
| 2001 | Andrews | 34 | fluvastatin | 64 | 477 |
| 2001 | Andrews | 34 | lovastatin | 42 | 476 |
| 2001 | Andrews | 34 | pravastatin | 20 | 462 |
| 2001 | Andrews | 34 | simvastatin | 39 | 468 |
| 2002 | GREACE | 35 | atorvastatin | 6 | 800 |
| 2002 | GREACE | 35 | placebo | 3 | 800 |
| 2002 | Davidson | 36 | placebo | 3 | 70 |
| 2002 | Davidson | 36 | simvastatin | 14 | 263 |
| 2002 | FLORIDA | 37 | fluvastatin | 30 | 265 |
| 2002 | FLORIDA | 37 | placebo | 37 | 275 |
| 2002 | LIPS | 38 | fluvastatin | 174 | 844 |
| 2002 | LIPS | 38 | placebo | 196 | 833 |
| 2002 | PROSPER | 39 | placebo | 116 | 1913 |
| 2002 | PROSPER | 39 | pravastatin | 107 | 2891 |
| 2002 | Olsson | 40 | rosuvastatin | 16 | 272 |
| 2002 | Olsson | 40 | atorvastatin | 12 | 140 |
| 2002 | Davidson | 41 | placebo | 7 | 132 |
| 2002 | Davidson | 41 | rosuvastatin | 10 | 259 |
| 2002 | Davidson | 41 | atorvastatin | 4 | 128 |
| 2002 | CHALLENGE | 42 | atorvastatin | 17 | 846 |
| 2002 | CHALLENGE | 42 | simvastatin | 10 | 848 |
| 2003 | Ballantyne | 43 | atorvastatin | 13 | 248 |
| 2003 | Ballantyne | 43 | placebo | 3 | 60 |
| 2003 | ADVOCATE | 44 | atorvastatin | 6 | 82 |
| 2003 | ADVOCATE | 44 | simvastatin | 2 | 76 |
| 2003 | Bruckert | 45 | fluvastatin | 13 | 607 |
| 2003 | Bruckert | 45 | placebo | 8 | 622 |
| 2003 | Kerzner | 46 | lovastatin | 10 | 220 |
| 2003 | Kerzner | 46 | placebo | 5 | 64 |
| 2003 | Melani | 47 | pravastatin | 3 | 205 |
| 2003 | Melani | 47 | placebo | 5 | 65 |
| 2003 | TREAT TO TARGET | 48 | atorvastatin | 20 | 552 |
| 2003 | TREAT TO TARGET | 48 | simvastatin | 14 | 535 |
| 2003 | HeFH | 49 | rosuvastatin | 16 | 436 |
| 2003 | HeFH | 49 | atorvastatin | 6 | 187 |
| 2003 | Mohler | 50 | atorvastatin | 16 | 240 |
| 2003 | Mohler | 50 | placebo | 10 | 114 |
| 2003 | Davidson | 51 | lovastatin | 21 | 501 |
| 2003 | Davidson | 51 | fluvastatin | 22 | 337 |
| 2003 | STELLAR | 52 | rosuvastatin | 9 | 480 |
| 2003 | STELLAR | 52 | atorvastatin | 25 | 641 |
| 2003 | STELLAR | 52 | simvastatin | 19 | 655 |
| 2003 | STELLAR | 52 | pravastatin | 11 | 492 |
| 2004 | CARDS | 53 | placebo | 145 | 1410 |
| 2004 | CARDS | 53 | atorvastatin | 122 | 1428 |
| 2004 | Bays | 54 | simvastatin | 31 | 622 |
| 2004 | Bays | 54 | placebo | 2 | 148 |
| 2004 | PREVENT IT | 55 | placebo | 22 | 431 |
| 2004 | PREVENT IT | 55 | pravastatin | 13 | 433 |
| 2004 | Durazzo | 56 | atorvastatin | 1 | 50 |
| 2004 | Durazzo | 56 | placebo | 0 | 50 |
| 2004 | Goldberg | 57 | placebo | 2 | 93 |
| 2004 | Goldberg | 57 | simvastatin | 7 | 349 |
| 2004 | ALLIANCE | 58 | atorvastatin | 75 | 1217 |
| 2004 | ALLIANCE | 58 | placebo | 3 | 1225 |
| 2004 | PCS | 59 | pravastatin | 5 | 54 |
| 2004 | PCS | 59 | placebo | 0 | 66 |
| 2004 | REVERSAL | 60 | pravastatin | 22 | 327 |
| 2004 | REVERSAL | 60 | atorvastatin | 21 | 327 |
| 2004 | DISCOVERY | 61 | rosuvastatin | 24 | 686 |
| 2004 | DISCOVERY | 61 | atorvastatin | 9 | 338 |
| 2004 | Schwatrz | 62 | rosuvastatin | 12 | 255 |
| 2004 | Schwatrz | 62 | atorvastatin | 6 | 128 |
| 2004 | Brown | 63 | rosuvastatin | 22 | 239 |
| 2004 | Brown | 63 | pravastatin | 11 | 118 |
| 2004 | Brown | 63 | simvastatin | 9 | 120 |
| 2005 | BELLES | 64 | atorvastatin | 43 | 305 |
| 2005 | BELLES | 64 | pravastatin | 21 | 309 |
| 2005 | DISCOVERY-Penta | 65 | rosuvastatin | 17 | 358 |
| 2005 | DISCOVERY-Penta | 65 | atorvastatin | 7 | 383 |
| 2005 | IDEAL | 66 | simvastatin | 186 | 4449 |
| 2005 | IDEAL | 66 | atorvastatin | 426 | 4439 |
| 2005 | CORALL | 67 | rosuvastatin | 9 | 131 |
| 2005 | CORALL | 67 | atorvastatin | 11 | 132 |
| 2005 | URANUS | 68 | rosuvastatin | 3 | 232 |
| 2005 | URANUS | 68 | atorvastatin | 7 | 233 |
| 2005 | COMETS | 69 | rosuvastatin | 4 | 165 |
| 2005 | COMETS | 69 | atorvastatin | 4 | 157 |
| 2005 | COMETS | 69 | placebo | 3 | 79 |
| 2006 | DISCOVERY-Alpha | 70 | rosuvastatin | 23 | 555 |
| 2006 | DISCOVERY-Alpha | 70 | atorvastatin | 14 | 382 |
| 2006 | SPARCL | 71 | atorvastatin | 415 | 2365 |
| 2006 | SPARCL | 71 | placebo | 342 | 2366 |
| 2006 | ASPEN | 72 | atorvastatin | 33 | 1211 |
| 2006 | ASPEN | 72 | placebo | 38 | 1199 |
| 2006 | PULSAR | 73 | rosuvastatin | 14 | 504 |
| 2006 | PULSAR | 73 | atorvastatin | 11 | 492 |
| 2006 | ARIES | 74 | rosuvastatin | 13 | 391 |
| 2006 | ARIES | 74 | atorvastatin | 10 | 383 |
| 2006 | STARSHIP | 75 | rosuvastatin | 11 | 357 |
| 2006 | STARSHIP | 75 | atorvastatin | 5 | 339 |
| 2006 | MERCURY II | 76 | rosuvastatin | 15 | 392 |
| 2006 | MERCURY II | 76 | atorvastatin | 19 | 798 |
| 2006 | MERCURY II | 76 | simvastatin | 25 | 803 |
| 2007 | METEOR | 77 | rosuvastatin | 79 | 702 |
| 2007 | METEOR | 77 | placebo | 22 | 282 |
| 2007 | SAGE | 78 | atorvastatin | 48 | 446 |
| 2007 | SAGE | 78 | pravastatin | 46 | 445 |
| 2007 | Kyeong | 79 | rosuvastatin | 2 | 60 |
| 2007 | Kyeong | 79 | atorvastatin | 3 | 57 |
| 2007 | ASTRONOMER | 80 | rosuvastatin | 25 | 134 |
| 2007 | ASTRONOMER | 80 | placebo | 26 | 135 |
| 2007 | CORONA | 81 | placebo | 302 | 2497 |
| 2007 | CORONA | 81 | rosuvastatin | 241 | 2514 |
| 2007 | Lewis | 82 | pravastatin | 11 | 163 |
| 2007 | Lewis | 82 | placebo | 16 | 163 |
| 2007 | ANDROMEDA | 83 | rosuvastatin | 15 | 248 |
| 2007 | ANDROMEDA | 83 | atorvastatin | 13 | 246 |
| 2007 | POLARIS | 84 | rosuvastatin | 22 | 432 |
| 2007 | POLARIS | 84 | atorvastatin | 27 | 439 |
| 2007 | DISCOVERY-Asia | 85 | rosuvastatin | 21 | 950 |
| 2007 | DISCOVERY-Asia | 85 | atorvastatin | 10 | 472 |
| 2007 | SOLAR | 86 | rosuvastatin | 15 | 542 |
| 2007 | SOLAR | 86 | atorvastatin | 20 | 544 |
| 2007 | SOLAR | 86 | simvastatin | 20 | 546 |
| 2007 | IRIS | 87 | rosuvastatin | 14 | 371 |
| 2007 | IRIS | 87 | atorvastatin | 7 | 369 |
| 2008 | GISSI-HF | 88 | rosuvastatin | 104 | 2285 |
| 2008 | GISSI-HF | 88 | placebo | 91 | 2289 |
| 2008 | ECLIPSE | 89 | rosuvastatin | 41 | 522 |
| 2008 | ECLIPSE | 89 | atorvastatin | 36 | 514 |
| 2008 | SUBARU | 90 | atorvastatin | 0 | 213 |
| 2008 | SUBARU | 90 | rosuvastatin | 8 | 214 |
| 2008 | DISCOVERY-Beta | 91 | rosuvastatin | 24 | 334 |
| 2008 | DISCOVERY-Beta | 91 | simvastatin | 7 | 170 |
| 2008 | Sdringola | 92 | placebo | 3 | 73 |
| 2008 | Sdringola | 92 | atorvastatin | 1 | 72 |
| 2009 | SPACE ROCKET | 93 | rosuvastatin | 20 | 633 |
| 2009 | SPACE ROCKET | 93 | simvastatin | 9 | 630 |
| 2009 | Ose | 94 | simvastatin | 4 | 219 |
| 2009 | Ose | 94 | pitavastatin | 21 | 638 |
| 2010 | CENTAURUS | 95 | rosuvastatin | 15 | 437 |
| 2010 | CENTAURUS | 95 | atorvastatin | 17 | 450 |
| 2010 | Acala | 96 | placebo | 0 | 70 |
| 2010 | Acala | 96 | pravastatin | 2 | 61 |
| 2011 | SATURN | 97 | atorvastatin | 48 | 519 |
| 2011 | SATURN | 97 | rosuvastatin | 45 | 520 |
| 2011 | Eriksson | 98 | pitavastatin | 9 | 236 |
| 2011 | Eriksson | 98 | simvastatin | 6 | 119 |
| 2011 | Gumprecht | 99 | pitavastatin | 8 | 275 |
| 2011 | Gumprecht | 99 | atorvastatin | 6 | 137 |
| 2011 | PATROL | 100 | atorvastatin | 13 | 101 |
| 2011 | PATROL | 100 | rosuvastatin | 10 | 100 |
| 2011 | PATROL | 100 | pitavastatin | 12 | 101 |
| 2012 | LUNAR | 101 | rosuvastatin | 26 | 499 |
| 2012 | LUNAR | 101 | atorvastatin | 25 | 257 |
