# Supplementary material for: CINeMA: An approach for assessing confidence in the results of a network meta-analysis
Source: PLoS Med. 2020 Apr 3;17(4):e1003082. doi: 10.1371/journal.pmed.1003082 (PMC7122720; doi:10.1371/journal.pmed.1003082)
Supplement: S1 Table — Odds ratios and their 95% confidence intervals are presented. The odds ratios presented in the upper triangle are the reciprocals of the odds ratios presented in the lower triangle. Odds ratios less than 1 favour the treatment specified in the row. (DOCX) [file pmed.1003082.s003.docx]

| **Atorvastatin** | 0.894  (0.637, 1.255) | 1.196  (0.868, 1.647) | 1.127  (0.637, 1.994) | 1.073  (0.890, 1.294) | 1.418  (1.126, 1.785) | 1.007  (0.848, 1.196) | 1.297  (1.065, 1.580) |
| --- | --- | --- | --- | --- | --- | --- | --- |
| 1.119  (0.797, 1.571) | **Fluvastatin** | 1.338  (0.915, 1.956) | 1.261  (0.655, 2.426) | 1.201  (0.879, 1.640) | 1.586  (1.109, 2.268) | 1.127  (0.787, 1.612) | 1.451  (1.011, 2.083) |
| 0.836  (0.607, 1.152) | 0.747  (0.511, 1.093) | **Lovastatin** | 0.942  (0.494, 1.797) | 0.897  (0.668, 1.206) | 1.185  (0.849, 1.655) | 0.842  (0.599, 1.184) | 1.085  (0.768, 1.532) |
| 0.887  (0.501, 1.570) | 0.793  (0.412, 1.526) | 1.061  (0.557, 2.023) | **Pitavastatin** | 0.952  (0.528, 1.718) | 1.258  (0.687, 2.305) | 0.893  (0.499, 1.598) | 1.151  (0.648, 2.043) |
| 0.932  (0.773, 1.123) | 0.833  (0.610, 1.138) | 1.114  (0.829, 1.498) | 1.050  (0.582, 1.895) | **Placebo** | 1.321  (1.070, 1.632) | 0.938  (0.759, 1.160) | 1.209  (0.960, 1.522) |
| 0.705  (0.560, 0.888) | 0.630  (0.441, 0.902) | 0.844  (0.604, 1.178) | 0.795 (0.434, 1.457) | 0.757  (0.613, 0.935) | **Pravastatin** | 0.710  (0.549, 0.918) | 0.915  (0.702, 1.193) |
| 0.993  (0.836, 1.179) | 0.888  (0.620, 1.270) | 1.188  (0.844, 1.671) | 1.119  (0.626, 2.002) | 1.066  (0.862, 1.317) | 1.408  (1.089, 1.821) | **Rosuvastatin** | 1.288  (1.024, 1.621) |
| 0.771  (0.633, 0.939) | 0.689  (0.480, 0.989) | 0.922  (0.653, 1.302) | 0.869  (0.489, 1.542) | 0.827  (0.657, 1.041) | 1.093  (0.839, 1.424) | 0.776  (0.617, 0.977) | **Simvastatin** |
