# Supplementary material for: CINeMA: An approach for assessing confidence in the results of a network meta-analysis
Source: PLoS Med. 2020 Apr 3;17(4):e1003082. doi: 10.1371/journal.pmed.1003082 (PMC7122720; doi:10.1371/journal.pmed.1003082)
Supplement: S2 Table — OR, odds ratio; SIDE, Separating Indirect from Direct Evidence. (DOCX) [file pmed.1003082.s004.docx]

| **Comparison** | **Direct OR** | **Indirect OR** | **Ratio of ORs** | **z-value** | **p-value** |
| --- | --- | --- | --- | --- | --- |
| Pravastatin versus rosuvastatin | 0.98 | 0.67 | 1.47 | 1.06 | 0.29 |
| Pravastatin versus simvastatin | 0.84 | 0.95 | 0.89 | -0.42 | 0.67 |
| Rosuvastatin versus simvastatin | 1.23 | 1.32 | 0.93 | -0.27 | 0.78 |
