# Supplementary material for: CINeMA: An approach for assessing confidence in the results of a network meta-analysis
Source: PLoS Med. 2020 Apr 3;17(4):e1003082. doi: 10.1371/journal.pmed.1003082 (PMC7122720; doi:10.1371/journal.pmed.1003082)
Supplement: S1 Text — (DOCX) [file pmed.1003082.s005.docx]

## Description of the data

Cipriani et al compared 18 commonly prescribed antidepressants, which were studied in 179 head-to-head randomized trials involving patients diagnosed with major/moderate depression [1]. The primary efficacy outcome was response measured as 50% reduction in the symptoms scale between baseline and 8 weeks of follow-up. According to the inclusion criteria specified in the protocol only studies at low or moderate risk of bias were included [2]. The methodological and statistical details presented in the published article and its appendix. Here, we will focus on how judgements about credibility of the results were derived. The network is presented below and the data is available in Mendeley Data (DOI:10.17632/83rthbp8ys.2).


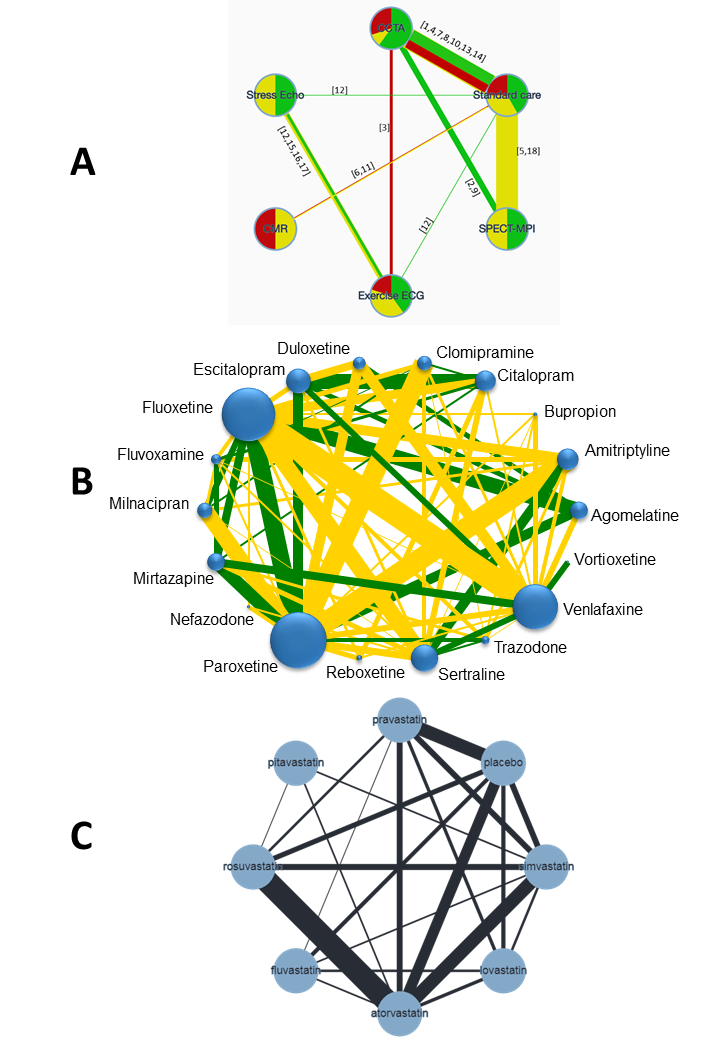


Network of randomised controlled trials comparing active antidepressants in patients with moderate/major depression. The colours of edges refer to the risk of bias; low (green), moderate (yellow) and red (high). The size of nodes is proportional to the number of studies examining each treatment.

# Within-study bias

We will focus on evaluating the results for three comparisons; amitriptyline vs milnacipran (one direct study at low and one at moderate risk of bias), mirtazapine versus paroxetine (three direct studies at low risk of bias and two at moderate) and amitriptyline vs clomipramine (no direct studies). The odds ratios for treatment response are presented below. We use this example to illustrate the use of sensitivity analysis and how it can inform the amount of contribution of studies at moderate and high risk of bias that we can tolerate.

Summary odds ratios from network meta-analysis comparing six antidepressants and sensitivity analyses excluding studies at moderate risk of bias.

| **Comparison** | **Response odds ratio [95% confidence interval]** | |
| --- | --- | --- |
|  | **All studies  (179 studies)** | **Studies at low risk of bias  (83 studies)** |
| Amitriptyline versus Milnacipran | 1.11 [0.85; 1.43] | 1.10 [0.77; 1.59] |
| Mirtazapine versus Paroxetine | 1.07 [0.88; 1.30] | 1.08 [0.83; 1.39] |
| Amitriptyline versus Clomipramine | 1.24 [0.97; 1.59] | 0.96 [0.59; 1.57] |

For the first two treatment comparisons in the table above, the contribution from studies at low risk of bias is more than 50%. Moreover, the sensitivity analysis excluding studies at moderate risk of bias provides results comparable to those obtained from all studies. Thus, one can derive the judgment of no concerns for amitriptyline versus milnacipran and mirtazapine versus paroxetine. However, the estimation of the relative treatment effect of amitriptyline versus clomipramine comes by more than 60% from studies at moderate risk of bias. Given also that the odds ratio from the sensitivity analysis is quite different from to the one obtained from all studies, we decided that there are some concerns for the amitriptyline versus clomipramine comparison.

#
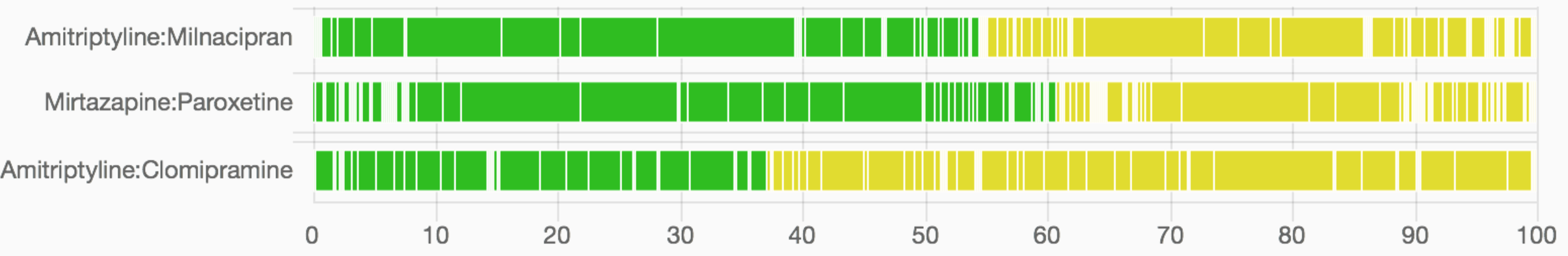


Risk of bias bars to decide about concerns due to within-study biases after considering the results from sensitivity analysis.

# Reporting bias

The literature search retrieved supplementary and unpublished information from clinical trial registries, regulatory agencies’ repositories and drug companies’ websites (particularly for the newest and most recently marketed antidepressants). Results from published and unpublished studies did not differ materially, no asymmetry was observed in the funnel plot [1] and meta-regression did not indicate an association between study precision and study odds ratio. However, the authors decided that they cannot completely rule out the possibility that some studies are missing because the field of antidepressant trials has been shown to be prone to publication bias. Consequently, the review team decided to assume that reporting bias was ‘suspected’ for all drug comparisons.

# Indirectness

Cipriani et al concluded that there is no indirectness in any of the studies included and that the distribution of modifiers was similar across studies and comparisons [1]. However, they decided to downgrade evidence about drugs that are poorly connected to the network. For example, vortioxetine was examined in a single study and consequently it was difficult to assess the comparability of effect modifiers in the comparisons with vortioxetine. Consequently, Cipriani et al. voiced concerns about indirectness for all comparisons with vortioxetine.

# Imprecision

In the network of antidepressants, the authors defined clinically important effects as an odds ratio smaller than 0.8 and larger than its reciprocal 1.25 [1]. We use this range of equivalence (0.8 to 1.25) in this example. We will concentrate on three comparisons, clomipramine versus fluvoxamine, citalopram versus venlafaxine and amitriptyline versus paroxetine. The 95% confidence interval of the odds ratio comparing clomipramine with fluvoxamine (0.75 to 1.32) includes clinically important effects in both directions, implying large uncertainty in which drug should be favored (‘major concerns’). The odds ratio for citalopram versus venlafaxine is 1.12 (95% confidence interval 0.90 to 1.39), favoring venlafaxine, but the interval includes values within the (0.8, 1) range. The verdict therefore is ‘some concerns’. Finally, the odds ratio of amitriptyline versus paroxetine is 0.96 (95% confidence interval 0.82 to 1.13) in favor of amitriptyline. Despite the fact that the estimate includes 1, it is not imprecise because the 95% confidence interval is within the range of equivalence (‘no concerns’).

Results from direct, indirect and mixed evidence along with confidence and prediction intervals and incoherence ratio of odds ratios for the network of antidepressants. Odds ratios lower than 1 favour the first treatment.

| **Comparison** | **Direct OR (95% CI)** | **Indirect OR (95% CI)** | **Ratio of ORs (95% CI)** | **NMA OR (95% CI)** | **95% PrI of NMA OR** |
| --- | --- | --- | --- | --- | --- |
| **Clomipramine versus Fluvoxamine** | 1.85  (0.65 to 5.27) | 0.96  (0.71 to 1.29) | 1.94  (0.65 to 5.73) | 0.99  (0.75 to 1.32) | (0.63 to 1.57) |
| **Citalopram versus Venlafaxine** | 1.72  (0.89 to 3.32) | 0.83  (0.66 to 1.04) | 2.08  (1.03 to 4.18) | 1.12  (0.90 to 1.39) | (0.74 to 1.70) |
| **Amitriptyline versus Paroxetine** | 1.07  (0.85 to 1.36) | 1.02  (0.82 to 1.27) | 1.05  (0.76 to 1.46) | 0.96  (0.82 to 1.13) | (0.65 to 1.42) |

NMA: network meta-analysis, OR: odds ratio, PrI: prediction interval, CI: confidence interval.

# Heterogeneity

In the antidepressants network, the estimated amount of heterogeneity is small $\left( \tau^{2}=0.03 \right)$. The prediction interval for clomipramine versus fluvoxamine does not add further uncertainty to clinical conclusions beyond that already represented by the confidence interval, so we have ‘no concerns’ about heterogeneity for that comparison. The prediction interval of citalopram versus venlafaxine extend into clinically important effects in both directions (0.74 to 1.70) while the confidence interval does not extend into values in favour of citalopram, thus suggesting potential implications of heterogeneity (‘some concerns’). We have ‘major concerns’ about the impact of heterogeneity for the comparison amitriptyline versus paroxetine, since the confidence interval lies entirely within the range of equivalence, whereas the prediction interval includes clinically important effects in favour of both treatments (0.65, 1.42).

Level of concern for three network meta-analysis odds ratios from the network of antidepressants for the domains imprecision, heterogeneity and incoherence.

| Comparison | Imprecision | Heterogeneity | Incoherence |
| --- | --- | --- | --- |
| Clomipramine versus Fluvoxamine | Major concerns | No concerns | No concerns |
| Citalopram versus Venlafaxine | Some concerns | Some concerns | Major concerns |
| Amitriptyline versus Paroxetine | No concerns | Major concerns | No concerns |

# Incoherence

In the network of antidepressants, the direct odds ratio comparing clomipramine with fluvoxamine is almost double the indirect odds ratio: the ratio of the two odds ratios (i.e., the inconsistency factor) is 1.94 (95% confidence interval 0.65 to 5.73). However, both direct and indirect estimates contain values that extend to clinically important values in both directions and the p-value is larger than 0.10. Thus, incoherence will not affect the interpretation of the NMA treatment effect: there are ‘no concerns’. In contrast, there are ‘major concerns’ regarding the confidence in the citalopram versus venlafaxine comparison: the direct odds ratio contains values within and above the range of equivalence while the indirect odds ratio includes values within and below the range of equivalence. The resulting estimated ratio of odds ratios is 2.08 (95% confidence interval 1.03 to 4.18) and the respective p-value of the SIDE test is 0.04. For the comparisons of amitriptyline versus paroxetine, the ratio of direct to indirect odds ratios is 1.05 (with 95% confidence interval (0.76, 1.46) and p-value 0.75) implying that the two sources of evidence are in agreement. Direct and indirect estimates are very close in terms of odds ratios, 95% confidence intervals and the range of equivalence and we therefore have ‘no concerns’ regarding incoherence for this particular comparison.

1. Cipriani A, Furukawa TA, Salanti G, Chaimani A, Atkinson LZ, Ogawa Y, et al. Comparative efficacy and acceptability of 21 antidepressant drugs for the acute treatment of adults with major depressive disorder: a systematic review and network meta-analysis. Lancet. 2018. doi:10.1016/S0140-6736(17)32802-7

2. Furukawa TA, Salanti G, Atkinson LZ, Leucht S, Ruhe HG, Turner EH, et al. Comparative efficacy and acceptability of first-generation and second-generation antidepressants in the acute treatment of major depression: protocol for a network meta-analysis. BMJ Open. 2016;6: e010919. doi:10.1136/bmjopen-2015-010919
